# Alcohol intoxication, but not hangover, differentially impairs learning and automatization of complex motor response sequences

**DOI:** 10.1038/s41598-021-90803-5

**Published:** 2021-06-15

**Authors:** Antje Opitz, Filippo Ghin, Jan Hubert, Joris C. Verster, Christian Beste, Ann-Kathrin Stock

**Affiliations:** 1 Cognitive Neurophysiology, Department of Child and Adolescent Psychiatry, Faculty of Medicine, TU Dresden, Schubertstrasse 42, 01309 Dresden, Germany; 2 Division of Pharmacology, Utrecht Institute for Pharmaceutical Sciences (UIPS), Utrecht University, Utrecht, The Netherlands; 3 Centre for Human Psychopharmacology, Swinburne University of Technology, Melbourne, Australia; 4 Biopsychology, Department of Psychology, School of Science, TU Dresden, Dresden, Germany

**Keywords:** Neuroscience, Psychology

## Abstract

Behavioral automatization usually makes us more efficient and less error-prone, but may also foster dysfunctional behavior like alcohol abuse. Yet, it has remained unclear whether alcohol itself causes the shift from controlled to habitual behavior commonly observed in alcohol use disorder (AUD). We thus investigated how the acute and post-acute effects of binge drinking affect the automatization of motor response sequences and the execution of automated vs. controlled motor response sequences. *N* = 70 healthy young men performed a newly developed automatization paradigm once sober and once after binge drinking (half of them intoxicated and half of them hungover). While we found no significant effects of alcohol hangover, acute intoxication (~ 1.2 ‰) had two dissociable effects: Firstly, it impaired the automatization of complex motor response sequence execution. Secondly, it eliminated learning effects in response selection and pre-motor planning processes. The results suggest that alcohol hangover did not affect controlled or automated processes, and disprove the assumption that alcohol intoxication generally spares or facilitates motor response sequence automatization. As these effects could be specific to the investigated explicit learning context, acute intoxication might potentially still improve the execution of pre-existing automatisms and/or the implicit acquisition of motor response sequence automatisms.

## Introduction

Binge drinking is a risky pattern of alcohol consumption observed worldwide, but is most prevalent in Europe, where it is most common among young adults (33.9% of all 20- to 24-year-olds)^1^. The US National Institute on Alcohol Abuse and Alcoholism (NIAAA) defines binge drinking as consuming at least four drinks for females and five drinks for males within a few hours, resulting in blood alcohol concentrations (BAC) of at least 0.8 ‰^2^. Similarly, the European School Survey Project on Alcohol and Other Drugs (ESPAD) Group defines binge drinking as consuming at least five drinks on one or more occasions within a 30-day period, where one drink comprises of around twenty milliliters of ethanol^3^. Aside from the acute intoxication effect, binge drinking may also have post-acute effects, as it often results in alcohol hangover (starting at 0.00 ‰). Both of these effects are known to change cognition and behavior^4–12^. In particular, acute intoxication has been shown to detrimentally interfere with top-down cognitive control, while automatic behavior seems rather unaffected in comparison^13–17^.

It is commonly assumed that in case of regular binge drinking, this imbalance between decreased controlled and preserved habitual behavior may lead to increasing difficulties in controlling one’s drinking behavior^18,19^, eventually increasing the risk of developing an alcohol use disorder (AUD)^20–22^. Further supporting this assumption, AUD patients have been reported to show deficits in behavioral control^23^ and increases in behavioral automaticity^24^. Yet, these findings largely refer to consumption-related behavior, so that it has remained unclear whether alcohol-associated changes are based on a general underlying mechanism. Furthermore, it has remained unclear whether the imbalance between controlled and automated behavior is actually caused by alcohol consumption, or merely a premorbid/comorbid phenomenon found in AUD patients.

In order to investigate whether alcohol causes this behavioral shift, one needs to account for the acute effect of alcohol (i.e., intoxication), but also for its aftermath (i.e., hangover), as they represent distinct phenomena which might contribute differently to the overall consequences of excessive alcohol consumption. While intoxication is defined as the presence of alcohol in the body (typically measured via blood or breath concentrations), hangover is defined as “the combination of negative mental and physical symptoms which can be experienced after a single episode of alcohol consumption, starting when blood alcohol concentration (BAC) approaches zero”^25^. Further stressing the functional distinction between acute intoxication and hangover, previous studies have demonstrated that these two states do not always have the same effect on cognitive control^26^, and may potentially even produce opposing effects on processes like information accumulation for response selection^27^. Although there is only little research on whether and how post-acute binge drinking effects modulate the balance between goal-directed and automated processes, a recent study suggested that alcohol hangover may not modulate the interplay of controlled vs. automated response processes^26^. Likewise, hungover participants did not seem to alter their application of goal-directed and/or habitual learning strategies^28^. Yet still, alcohol hangover has been shown to impair cognitive control functions^29–33^, which could promote a relative advantage of response automatization as well as poor behavioral choices beyond acute intoxication, like the habitual continuation of drinking.

While single actions (like drinking a bottle of beer) may of course become habitual, it is much more likely that complex action sequences, which consist of several single actions (e.g., going to the fridge, opening the door, grabbing a drink, opening the bottle, and then finally consuming it), are eventually automated^34^. But while habitual action sequences play an important role in AUD and other dysfunctional behavior^35–37^, surprisingly little is known about how binge drinking, a factor known to increase AUD vulnerability^38^, affects the balance between the automated vs. controlled execution of action sequences^39^. Given the prevalent focus on directly addiction-related aspects of alcohol consumption in research, it has also rarely been investigated whether alcohol-unrelated action sequences are affected as well. Given that this would be needed to identify a general shift in cognitive functioning (rather than just proving altered consumption), it is important to focus on processes, which are involved in planning and execution of motor response sequences^40^. If the same motor response sequence is repeatedly executed and/or rewarded, increasingly stronger stimulus–response (S-R) associations are established, which ultimately results in the automatization of the given motor response sequence. Generally, the automatization of motor response sequences is a highly efficient way of prompting and organizing various behaviors^41,42^, but at the price that automatization leads to desensitization towards outcome devaluation^43–45^. The latter may give rise to dysfunctional behavior like excessive drinking, as behavior will then be maintained despite negative outcomes^39,43,44,46^. In the long run, this could contribute to the manifestation of dysfunctional behaviors, as seen in AUD. In order to investigate alcohol as a causal factor in the shift from controlled to habitual behavior, and to furthermore investigate whether this represents a shift in general cognitive functioning (i.e., independent of consumption-related behavior), we focused on how the acute and post-acute effects of binge drinking affect the acquisition of new automated motor response sequences and the execution of automated vs. controlled motor response processes in general.

To investigate these research questions, we developed a new paradigm that assesses the strength with which motor response sequences are automatized as the relative performance advantage over non-automatized (i.e., top-down controlled) performance. More specifically, this is achieved by comparing “control” task blocks to “automatization” task blocks. In the control blocks, motor response sequence generation requires top-down controlled processes due to the random generation of required response sequences. In automatization blocks, motor response sequence generation becomes automatized over the course of the experiment due to a high-frequency repetition of the same response sequence. We therefore expected that performance would be better in automatization blocks than in control blocks. Furthermore, this performance gap should increase from the first to the last task block (i.e., over the course of the experiment) as increasing motor response sequence automatization benefits behavior. In contrast to common motor sequence tasks (in which a single stimulus triggers the entire motor sequence), our paradigm investigates sequences of S-R associations, as a distinct stimulus triggers a single motor response. Eventually, multiple single motor responses form a motor response sequence. Based on previous findings of rather selective control deficits during acute and post-acute binge drinking^12,13,15,16,29,30^, we hypothesized that compared to sober performance, there should be alcohol-induced performance impairments in the control blocks, while performance should be relatively preserved in the automatization blocks. As a consequence, the performance gap between controlled and automated motor response sequence generation should be greater during acute intoxication and alcohol hangover than during sobriety. Additionally, the alcohol-related result pattern should be more pronounced during acute intoxication than during alcohol hangover. As behavioral measures reflecting overall performance, we assessed response accuracy and the duration of the entire response from stimulus onset to the last response (entire sequence duration / ESD). The latter was further subdivided into the time from stimulus onset to the first response to obtain a measure reflecting the cognitive processes involved in planning and selection of the motor response sequence (RT1) and into the time from the first to last motor response to obtain a measure reflecting processes involved in the coordination and execution of the motor response sequence (MSD). To this end, we subjected healthy young men to this paradigm in a mixed crossover study design, where each participant was either assigned to the alcohol hangover or the acute alcohol intoxication group. In both groups, we yielded for an average high-dose intoxication level of approximately 1.2 ‰. Both alcohol manipulation groups were experimentally intoxicated and tested twice in a counterbalanced order, that is once at a sober and once at an alcohol appointment (i.e., either intoxicated or hungover).

Taken together, we investigated how different effects of binge drinking, that is acute intoxication and alcohol hangover, affect the automatization of complex motor response sequences as well as the relative difference between controlled and automated motor response sequences. This is of particular importance as a potential imbalance towards increased automated and decreased controlled behavior during binge drinking could foster dysfunctional behavior in everyday life.

## Results

### Sample characteristics

*N* = 76 men were initially recruited and tested (mean age 23.2 ± 3.3 years old). Out of these, *n* = *6* had to be excluded from the analyses for the following reasons: One hangover group participant had a residual BrAC of 0.45 ‰ at the beginning of the hangover appointment and could not spend the required four to five hours waiting time until his BrAC had returned to zero. Three intoxication group participants had to be excluded due to poor performance during acute intoxication (accuracy below 50% in at least one of the four condition combinations). One intoxication group participant had to be excluded due to technical problems on the intoxication appointment, and another participant of that group had to be excluded due to very slow responses during intoxication (which resulted in the exclusion of more than 50% of the trials when applying the exclusion criteria described in the “Statistical analyses” section). Eventually, the data of *n* = 70 participants entered statistical analyses. *N* = 34 participants were in the intoxication group and *n* = 36 participants were in the hangover group. In both groups, *n* = 18 had their sober appointment before their alcohol appointment. *N* = 18 hangover group participants and *n* = 16 intoxication group participants had their alcohol appointment before their sober appointment. Table 1 provides statistical comparisons of sociodemographic characteristics and questionnaire scores between both alcohol groups as well as their group-specific alcohol-related data.

**Table 1:** Comparison of sociodemographic characteristics, questionnaire scores and alcohol-related data between both groups.

Characteristic	**Group**		*p*_*Difference*_
	Intoxicated (*n* = 34)	Hungover (*n* = 36)	
Age in years	22.74 ± 0.63 (18–30)	23.11 ± 0.45 (19–28)	0.368
Height in cm	182.18 ± 1.22 (168–195)	181.83 ± 0.95 (170–195)	0.825
Weight in kg	76.75 ± 1.76 (57–98)	77.24 ± 1.68 (63–105)	0.909
Hours of sport per week	4.41 ± 0.44 (0–12)	4.50 ± 0.56 (0–16)	0.745
Cigarettes smoked per day	0.37 ± 0.23 (0–7)	0.76 ± 0.36 (0–10)	0.159
BDI score	2.84 ± 0.57 (0–11)	3.44 ± 0.68 (0–19)	0.518
AUDIT score	6.94 ± 0.53 (3–14)	10.19 ± 0.57 (5–19)	< 0.001**
BrAC 30 min after end of consumption	1.17 ± 0.04 (0.64–1.69)	1.32 ± 0.03 (1.05–1.69)	0.002**
BrAC 60 min after end of consumption	1.16 ± 0.03 (0.88–1.57)	1.25 ± 0.02 (1.01–1.56)	0.030*
BrAC 90 min after end of consumption	1.00 ± 0.02 (0.73–1.37)	1.15 ± 0.02 (0.91–1.40)	< 0.001**
BrAC 120 min after end of consumption	0.93 ± 0.03 (0.66–1.28)	1.08 ± 0.03 (0.83–1.43)	< 0.001**
Individual alcohol amount indicated in grams﻿	90.29 ± 1.35 (75–108)	120.71 ± 1.66 (106–149)	< 0.001**
Alcohol consumption duration in minutes	31.64 ± 1.54 (20–57)	181.75 ± 4.18 (111–243)	< 0.001**

All values are reported as means ± standard error of the mean and the range is given in parentheses. The comparisons were performed with uncorrected independent t-tests or Mann–Whitney U-tests (in case of non-normal distributions). Both groups differed in their AUDIT score, which most likely resulted from the application of different AUDIT inclusion criteria during recruiting. Both groups also differed in their alcohol-related data as the experimental protocols systematically differed between the two groups (e.g., alcohol amount, consumption duration, type of alcohol, abstaining from meals before the appointment). *BDI* Beck depression inventory; *AUDIT* alcohol use disorders identification test; *BrAC* breath alcohol concentration; *p < .05, **p < .01.

### Comparability between the two samples

Given that previous studies suggested potential effects of drinking habits onto cognitive performance^22,47^, we wanted to make sure that the two experimental groups were indeed comparable despite some differences in their everyday drinking habits (AUDIT scores, please refer to Table 1 for details and the “Participants” section for the underlying reason). Therefore, we correlated the AUDIT score of all included participants with their behavioral performance for the entire sequence duration (ESD) measure and for the accuracy measure. None of these correlations reached significance (all r ≤ 0.190; *p* ≥ 0.115) and add-on Bayesian analyses provided moderate to strong evidence for the null hypothesis (no relevant correlation), as all BF_01_ > 3. We then also compared the behavioral performance at the sober appointment across alcohol manipulation groups using independent *t*-tests for the entire sequence duration (ESD) measure and Mann–Whitney-*U* tests for accuracy measure. We found no significant group differences for both the ESD (all *t* ≤ 1.759; all *p* ≥ 0.083) and accuracy (all *U* ≤ 682; all *p* ≥ 0.213). Additional Bayesian independent samples *t*-tests for all of these measures yielded BF_01_ > 1 for all behavioral parameters, thus showing that the null hypothesis (assumption of no group differences) was to be preferred over the alternative hypothesis in all cases. Taken together, this strongly suggests that drinking habits did not modulate performance and that the two groups did not perform differently at the sober (“baseline”) assessment. They should therefore be considered sufficiently comparable despite slightly different recruitment criteria.

For the hangover group, we additionally assessed subjective ratings of sleep duration, and hangover symptoms at both the sober and hangover appointment (fur further details, please see Supplement). The rating of overall hangover severity was assessed with a single item, which is considered to be a better and more accurate assessment than composite questionnaire scores^48^. It was significantly higher during the hungover appointment (3.75 ± 0.4) than during the sober appointment (0 ± 0) (*Z* = 5.108; *p* < 0.001), thus indicating a successful induction of alcohol hangover in this group. Of note, similar overall hangover severity scores have been reported in other experimental studies^28,49,50^, as well as in naturalistic studies where participants could drink ad libitum^51–54^.

### Task effects (irrespective of alcohol)

**Figure 1:**
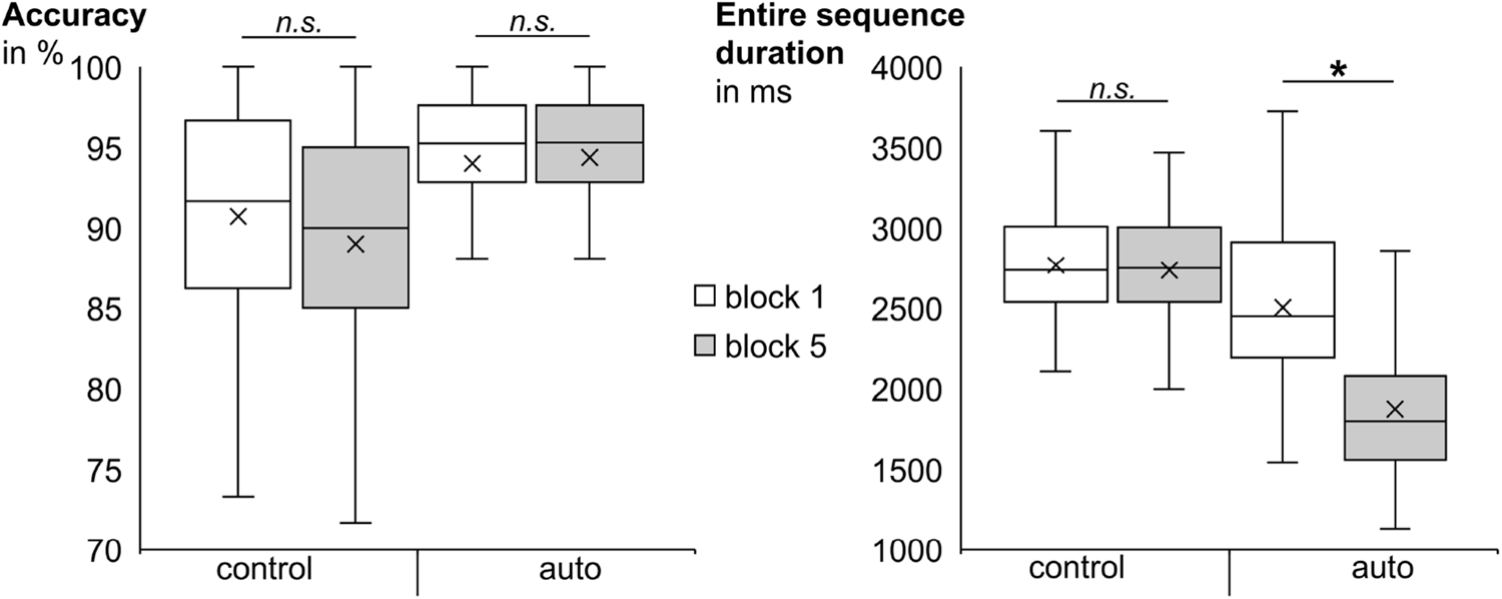
Illustration of the task effects in accuracy (in percent, left graph) and entire sequence duration (ESD, i.e., the time between stimulus onset and the last response in milliseconds, right graph). We observed significantly more accurate and faster responses in the auto condition than in control condition. For ESDs, we also observed significantly shorter durations in block 5 than in block 1. Most importantly, the size of the learning effect (block 1 minus block 5) was significantly larger in the auto condition (where automatization of the entire motor sequence was possible over the course of the experiment), than in the control condition (where this automatization was not possible). *n.s.* = non-significant; * = p < 0.001.

To demonstrate that and how the newly developed task assesses (motor response sequence) automatization effects, the following paragraph exclusively reports main effects and interactions of the task manipulations (i.e., disregarding all main and interaction effects of alcohol manipulation group and alcohol administration). The accuracy and ESD data for the task effects is visualized in Fig. 1.

Regarding accuracy, there was a main effect of condition (*F*_(1,68)_ = 59.475; *p* < 0.001; *η*^*2*^_*p*_ = 0.467), which showed better performance in auto trials (94.1% ± 0.5) than in control trials (89.8% ± 0.6). This performance improvement in trials where motor response sequence automatization was possible clearly demonstrates beneficial automatization effects. In addition, the interaction between condition and block showed a trend towards significance (*F*_(1,68)_ = 3.986; *p* = 0.050; *η*^*2*^_*p*_ = 0.055). However, separate post hoc comparisons showed no significant differences between block 1 and block 5 in neither the control nor the auto condition (all *t* ≤ 1.910; all *p* ≥ 0.060). Given the lack of significance for the interaction effect as well as the lack of significant block effects in both conditions, we refrained from further post hoc analyses. The main effect of block was non-significant (*F*_(1,68)_ = 1.177; *p* = 0.282).

The results of the mixed ANOVA for the ESD measure (i.e., the time interval between stimulus onset and last response in trials where all four responses were entered correctly and in the correct order) are summarized in Table 2. There was a main effect of condition, as ESDs were significantly shorter in auto trials (2228 ms ± 56) than in control trials (2797 ms ± 38). Like the condition effect found in the accuracy measure, this performance improvement in trials where motor response sequence automatization was possible clearly demonstrates beneficial automatization effects. Additionally, the main effect of block demonstrated a general learning effect, as ESDs were shorter in block 5 (2345 ms ± 44) than in block 1 (2681 ms ± 50). Furthermore, the interaction between condition and block reached significance (see Fig. 1). Separate post hoc comparisons for the two conditions showed that the participants got significantly faster from block 1 (2542 ms ± 63) to block 5 (1905 ms ± 62) in the auto condition (*t*_(69)_ = 14.459; *p* < 0.001), but not in the control condition (*t*_(69)_ = 1.151; *p* = 0.254). Thus, the learning effect (block 1 minus block 5) was significantly larger in the auto condition, where automatization of the entire motor sequence was possible over the course of the experiment (637 ms ± 44), than in the control condition, where automatization of the entire motor sequence was not possible (35 ms ± 31) (*t*_(69)_ =|12.928|; *p* < 0.001). This demonstrates that as intended, effective automatization took place in the automatization condition, but not in the control condition.

**Table 2:** Main and interaction effects of the mixed 2 × 2 × 2 × 2 ANOVA for entire sequence duration.

	F	df_effect_	df_error_	p	η^2^_p_
**Task effects**					
Condition	272.811	1	68	< .001 **	0.800
Block	124.972	1	68	< .001 **	0.648
Condition × block	165.163	1	68	< .001 **	0.708
**Alcohol effects**					
Alcohol manipulation group	10.600	1	68	0.002 **	0.135
Alcohol administration	3.369	1	68	0.071	0.047
Alcohol administration × alcohol manipulation group	10.920	1	68	0.002 **	0.138
Condition × alcohol manipulation group	0.441	1	68	0.509	0.006
Block × alcohol manipulation group	1.097	1	68	0.299	0.016
Alcohol administration × condition	1.511	1	68	0.223	0.022
Alcohol administration × condition × alcohol manipulation group	6.551	1	68	﻿0.013 *	0.088
Alcohol administration × block	1.414	1	68	0.239	0.020
Alcohol administration × block × alcohol manipulation group	9.758	1	68	0.003 **	0.125
Condition × block × alcohol manipulation group	0.441	1	68	0.509	0.006
Alcohol administration × condition × block	0.007	1	68	0.932	0.000
Alcohol administration × condition × block × alcohol manipulation group	2.052	1	68	0.157	0.029

*p < .05; **p < .01.

### Alcohol effects on the ESD measure

As the ESD reflected learning and automatization better than the accuracy measure in the task effect analyses, the alcohol-related analyses focused on the ESD. A graphic depiction of alcohol effects in each single condition and group can be found in the Supplementary Material.

In the ESD ANOVA, we found a main effect of alcohol manipulation group (see Table 2). As ESDs were longer in the intoxication group (2658 ms ± 64) than in the hangover group (2368 ms ± 62), and we had initially not found significant group differences in the sober assessment, this most likely evidences that performance was more strongly impaired by intoxication than by hangover. Additionally, significant effects were found for the interactions of alcohol administration x alcohol manipulation group, alcohol administration x condition x alcohol manipulation group, and alcohol administration x block x alcohol manipulation group. In the following two text sections, we separately report the post hoc findings of these two three-way interactions. To further investigate whether these specific alcohol effects observed for the ESD measure were more strongly based on response selection and planning, or on motor response execution, we repeated the four factor mixed ANOVA with the RT1 measure (i.e., the time passed between stimulus onset and the first motor response) and with the MSD measure (i.e., the time passed between the first and last response). In order to keep the results section concise, we only report the outcomes of the two three-way interactions of interest that were found to be relevant for the ESD measure.

#### Alcohol effects on the automated response execution of motor response sequences

**Figure 2:**
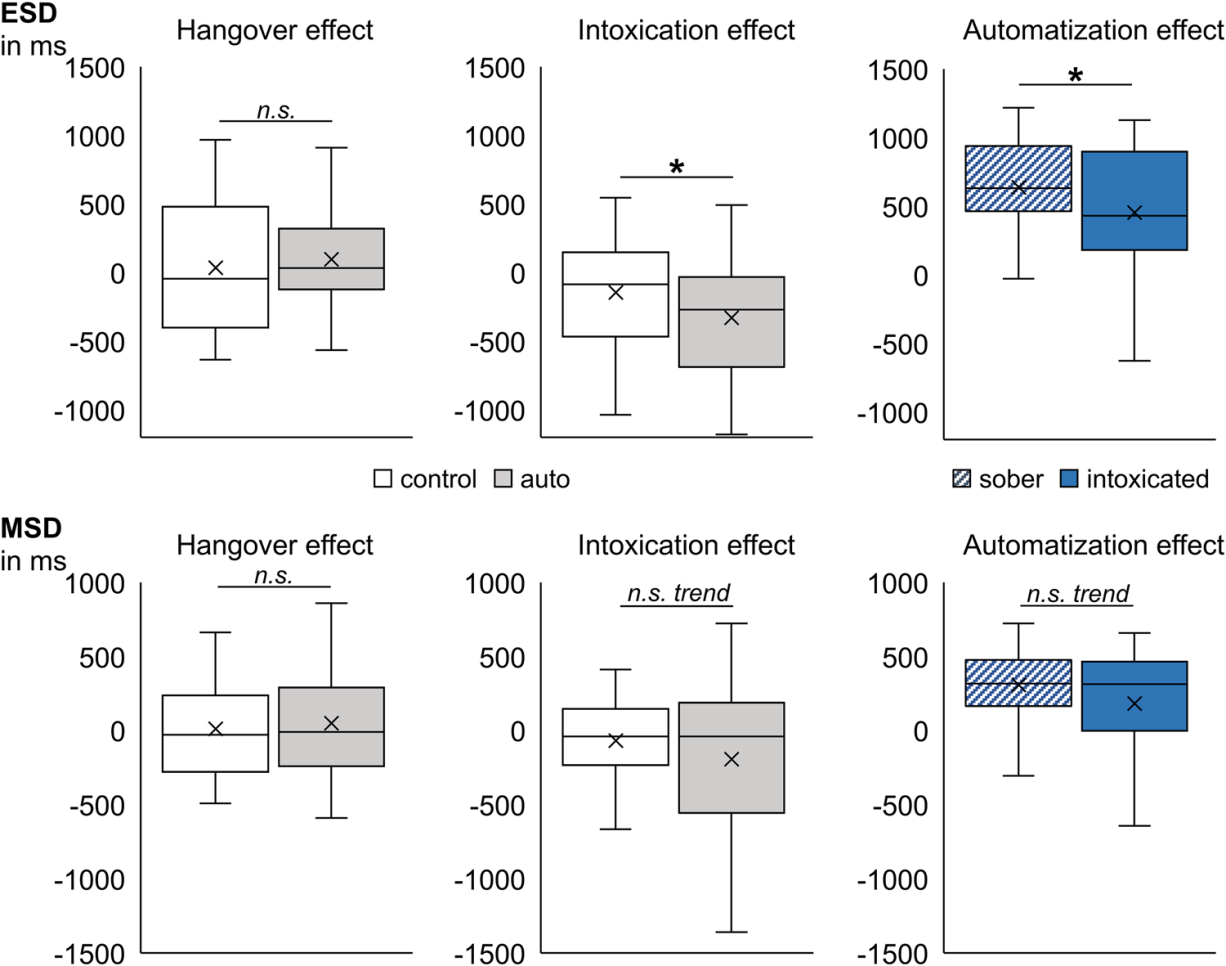
Illustration of the three-way interaction of alcohol administration x condition x alcohol manipulation group found for the entire sequence duration (ESD in milliseconds; upper graph) and for the motor sequence duration (MSD in milliseconds; lower graph). While there were no overall hangover-induced effects on task conditions, intoxication-induced impairments (i.e., the difference between sober and intoxicated performance) were significantly larger in the auto condition than in the control condition for the ESD measure and showed a corresponding trend for the MSD measure. Likewise, the size of the ESD automatization effect (i.e., the difference between control and auto condition) was significantly smaller at the intoxicated appointment than at the sober appointment and showed a corresponding trend for the MSD measure. This suggests that the intoxication-induced deficits in motor response sequence automatization arose mainly from intoxication-related impairments in the automatization of motor response execution processes, but not response selection and planning, as these ESD effects were observed for the MSD, but not the RT1 measure.

For the interaction of alcohol administration x condition x alcohol manipulation group in the ESD measure (see Table 2 and Fig. 2), we conducted separate post hoc tests for each alcohol manipulation group. The post hoc ANOVA for the hangover group showed no significant interaction between alcohol administration and condition (*F*_(1,35)_ = 0.917; *p* = 0.345). That is, alcohol hangover had no significant effect on the combination of task conditions, as compared to sobriety. In the post hoc ANOVA for the intoxication group, we obtained a significant interaction between alcohol administration and condition (*F*_(1,33)_ = 6.930; *p* = 0.013; *η*^*2*^_*p*_ = 0.174). A post hoc dependent samples test revealed that auto condition ESDs were longer at the intoxicated appointment (2549 ms ± 116) than at the sober appointment (2220 ms ± 89) (*t*_(33)_ =|4.227|; *p* < 0.001). Control condition ESDs were also longer at the intoxication appointment (3003 ms ± 66) than at the sober appointment (2860 ms ± 68) (*t*_(33)_ =|2.065|; *p* = 0.047). Yet, the size of the intoxication effect (sober minus intoxicated) was significantly larger in the auto condition (− 329 ms ± 78) than in the control condition (− 142 ms ± 69) (*t*_(33)_ =|2.633|; *p* = 0.013). This demonstrates stronger intoxication-induced impairments in the auto condition (where automatization of the entire motor sequence was possible), than in the control condition (where this automatization was not possible). As a consequence thereof, we found that the automatization effect (control minus auto) had become significantly smaller during acute intoxication (454 ms ± 79) as compared to the sober appointment (640 ms ± 57) (*t*_(33)_ =|2.633|; *p* = 0.013). To further explore the three-way interaction of administration x condition x alcohol manipulation group, we ran add-on independent samples tests that compared the two alcohol manipulation groups at the alcohol appointment (as these comparisons had not yielded any﻿ group differences for the sober condition, see section "Comparability between the two samples"). We observed longer ESDs in both auto and control condition at the intoxicated appointment (auto = 2549 ms ± 116; control = 3003 ms ± 66) compared to the hungover appointment (auto = 2022 ms ± 65; control = 2647 ms ± 60) (*U*_auto_ = 912; *p* < 0.001; *U*_control_ = 914.5; *p* < 0.001). However, it should be noted that performance was always better in the auto condition than in the control condition (as indicated by the significant main effect of condition in the separate post hoc ANOVAs of both groups [*F*_hangover(1,35)_ = 246.339; *p* < 0.001; *η*^2^_*p*_ = 0.876; *F*_intoxication(1,33)_ = 86.864; *p* < 0.001; *η*^2^_*p*_ = 0.725]). This indicates that automatization took place in both alcohol states, even though acute intoxication was more detrimental to performance than being hungover.

Regarding the RT1 and MSD measure, the three-way interaction of alcohol administration x condition x alcohol manipulation group was non-significant in the RT1 ANOVA (*F*_(1,68)_ = 0.992; *p* = 0.323), but significant in the MSD ANOVA (*F*_(1,68)_ = 4.852; *p* = 0.031; *η*^*2*^_*p*_ = 0.067) (please see Fig. 2). We conducted post hoc tests for this three-way interaction in the MSD measure in the same way as we had done for the ESD measure. Matching the ESD findings, there was no interaction of alcohol administration x condition in the hangover group (*F*_(1,35)_ = 1.419; *p* = 0.242). In the post hoc ANOVA for the intoxication group, the interaction of alcohol administration x condition did not reach significance (*F*_(1,33)_ = 3.337; *p* = 0.077). As this interaction effect was however significant in ESDs and showed a non-significant trend in MSDs, we performed an add-on Bayesian analysis as suggested by Masson^55^ in order to determine whether the null or alternative hypothesis of the intoxication effect was more likely. This yielded substantial positive evidence for the alternative hypothesis^56^, given the obtained data (P_BIC_(H1|D) = 93.1%; BF_01_ < 0.1). We thus decided to perform further post hoc analyses. Post hoc dependent samples tests revealed that MSDs were not significantly longer at the intoxicated appointment than at the sober appointment in either the auto or the control condition (*Z*_*auto*_ =|1.547|; *p* = 0.122; *t*_*control(33)*_ =|1.438|; *p* = 0.160). The size of the intoxication effect (sober minus intoxicated) was not significantly larger in the auto condition (− 192 ms ± 91) than in the control condition (− 67 ms ± 46), but showed a trend (*t*_*(33)*_= |1.827|; *p* = 0.077), which suggests a trend towards slightly stronger intoxication-related impairments in the auto condition (where automatization of motor execution was possible) compared to the control condition (where it was not possible). While the automatization effect (control minus auto) was smaller during acute intoxication (184 ms ± 65) as compared to the sober appointment (310 ms ± 38) on the descriptive level, the Wilcoxon-signed rank test did not reach significance (*Z* = |1.325|; *p* = 0.185). Further add-on analyses comparing the two alcohol manipulation groups at the alcohol appointment showed no significant differences in MSDs for neither auto condition (*U* = 723; *p* = 0.192) nor control condition (*t*_*(68)*_ =|1.951|; *p* = 0.055). However, it should be noted that performance was always better in the auto condition than in the control condition (as indicated by the main effect of condition in the separate post hoc ANOVAs of both groups [*F*_hangover(1,35)_ = 144.739; *p* < 0.001; *η*^2^_*p*_ = 0.805; *F*_intoxication(1,33)_ = 36.691; *p* < 0.001; *η*^2^_*p*_ = 0.526]), which indicates that the automatization of motor response execution still took place in both alcohol states.

#### Alcohol effects on response selection and planning-related learning

**Figure 3:**
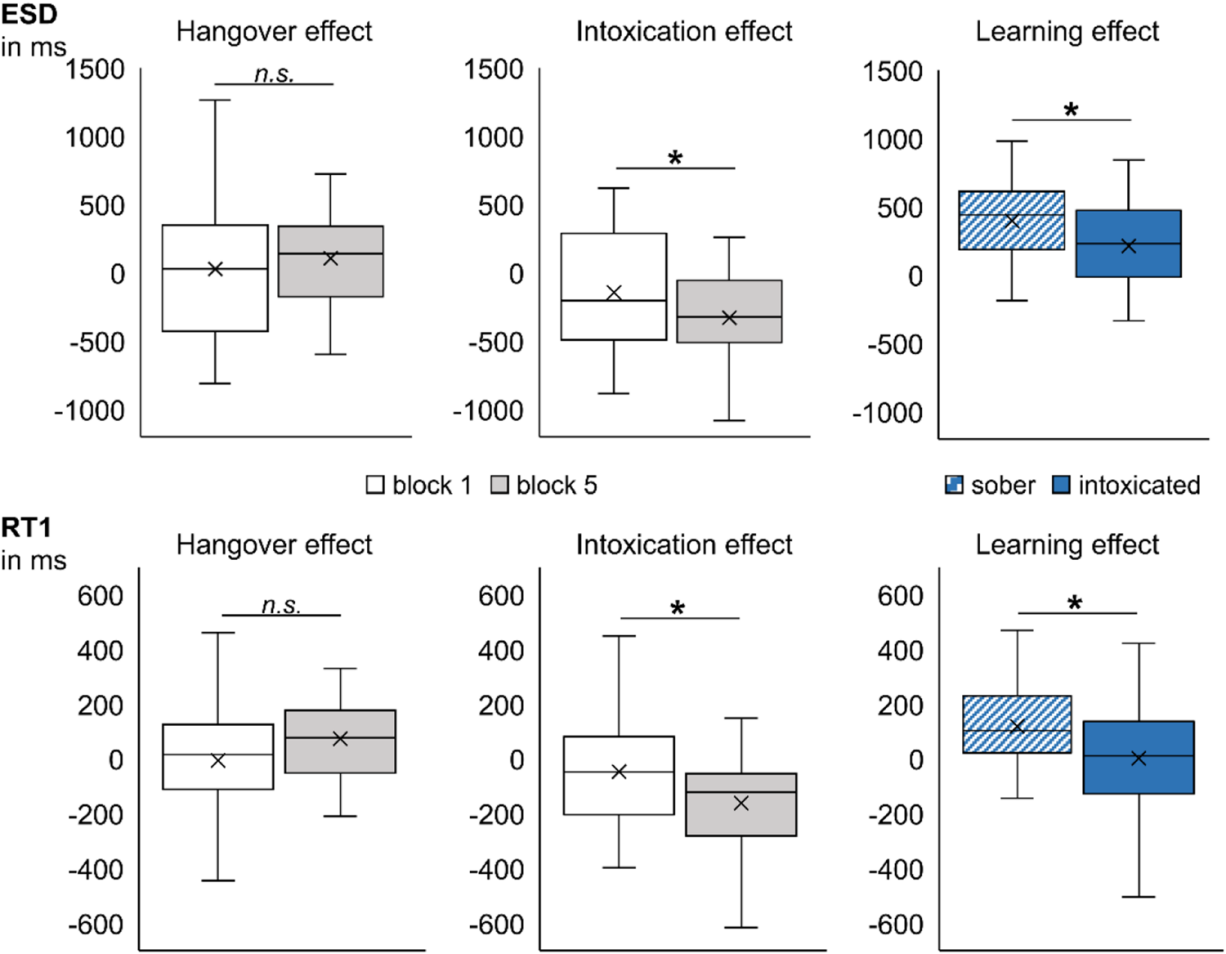
Illustration of the three-way interaction of alcohol administration x block x alcohol manipulation group found for the entire sequence duration (ESD in milliseconds; upper graph) and for the first motor response time (RT1 in milliseconds; lower graph). While there were no overall hangover-induced effects on block number, intoxication-induced impairments (i.e., the difference between sober and intoxicated performance) were significantly larger in block 5 than in block 1 for both depicted measures. The size of the learning effect (i.e., the difference between block 1 and block 5) was significantly smaller at the intoxicated appointment than at the sober appointment. This allows for the conclusion that the deficits in learning motor response sequences generation arose mainly from intoxication-induced impairments in response selection and planning processes, but not from motor response execution processes, as these ESD effects were only observed in the RT1, but not in the MSD measure.

For the interaction of alcohol administration x block x alcohol manipulation group in the ESD measure (please see Table 2 and Fig. 3), we conducted separate post hoc tests for the two alcohol manipulation groups. While the interaction of alcohol administration x block was not significant in the post hoc ANOVA for the hangover group (*F*_(1,35)_ = 1.757; *p* = 0.194), it was significant in the post hoc ANOVA for the intoxication group (*F*_(1,33)_ = 10.074; *p* = 0.003; *η*^*2*^_*p*_ = 0.234). Post hoc dependent sample tests showed that in block 5, ESDs were significantly longer during intoxication (2669 ms ± 89) than during sobriety (2343 ms ± 69) (*t*_(33)_ =|5.548|; *p* < 0.001). This effect could not be found in block 1 (*t*_(33)_ =|1.797|; *p* = 0.082). The size of the intoxication effect (sober minus intoxicated) was significantly larger in block 5 (− 326 ms ± 59) than in block 1 (− 145 ms ± 80) (*t*_(33)_ =|3.174|; *p* = 0.003). Furthermore, we found that the learning effect (block 1 minus block 5) had become significantly smaller during acute intoxication (213 ms ± 58) as compared to the sober appointment (395 ms ± 56) (*t*_(33)_ =|3.174|; *p* = 0.003). Taken together, this demonstrates intoxication- induced learning impairments that seemed to occur irrespective of automatization (as the factor of condition did not play any role for this interaction). To further investigate the interaction of administration x block x alcohol manipulation group, we ran add-on independent samples tests that compared the two alcohol manipulation groups at the alcohol appointment (as these comparisons had not yielded any group differences for the sober condition, see section "Comparability between the two samples"). For both blocks, we observed longer ESDs at the intoxicated appointment (block 1 = 2882 ms ± 91; block 5 = 2669 ms ± 89) as compared to the hungover appointment (block 1 = 2538 ms ± 69; block 5 = 2130 ms ± 52) (*t*_block1(68)_ =|3.021|; *p* = 0.004; *U*_block5_ = 1024; *p* < 0.001). In short, this indicates that intoxication impairs learning processes related to the generation of complex motor response sequences to a much stronger degree than hangover.

Regarding the RT1 and MSD measure, the three-way interaction of alcohol administration x block x alcohol manipulation group was non-significant in the MSD ANOVA (*F*_(1,68)_ = 1.212; *p* = 0.275), but significant in the RT1 ANOVA (*F*_(1,68)_ = 10.949; *p* = 0.002; *η*^*2*^_*p*_ = 0.139) (please see Fig. 3). We conducted post hoc tests for this three-way interaction in the RT1 measure in the same way as we had done for the ESD measure. Matching the ESD findings, there was no interaction of alcohol administration x block in the hangover group (*F*_(1,35)_ = 3.518; *p* = 0.069), while the post hoc ANOVA for the intoxication group revealed a significant interaction of alcohol administration x block (*F*_(1,33)_ = 8.051; *p* = 0.008; *η*^2^_*p*_ = 0.196﻿). Post hoc dependent samples tests showed that in block 5, the RT1 was slower during intoxication (1398 ms ± 48) than during sobriety (1237 ms ± 39) (*Z* = |4.163|; *p* < 0.001). This effect could not be found in block 1 (*t*_(33)_ =|1.325|; *p* = 0.194). The size of the intoxication effect (sober minus intoxicated) was significantly larger in block 5 (161 ms ± 32) than in block 1 (45 ms ± 34) (*t*_(33)_ =|2.837|; *p* = 0.008). Furthermore, we found that the learning effect (block 1 minus block 5) had become significantly smaller during acute intoxication (4 ms ± 31) as compared to the sober appointment (119 ms ± 32) (*t*_(33)_ =|2.837|; *p* = 0.008). This demonstrates intoxication-induced learning impairments that seemed to occur irrespective of automatization. Add-on analyses of alcohol manipulation group comparisons at the alcohol appointment yielded slower RT1s in both block 1 and block 5 at the intoxicated appointment (block 1 = 1402 ms ± 43; block 5 = 1398 ms ± 48) compared to the hungover appointment (block 1 = 1231 ms ± 31; block 5 = 1086 ms ± 27) (*t*_block1(68)_ =|3.228|; *p* = 0.002; *t*_block5(68)_ =|5.743|; *p* < 0.001). Taken together, this indicates that acute intoxication seems to eradicate any learning effects with respect to response selection and planning.

#### Summary of alcohol effects

To summarize, we did not find a significant interaction of all included factors. Instead, we found two different, seemingly independent alcohol-induced effects, which seem to arise from different sub-processes. Firstly, we found that alcohol intoxication affected the automatization of a complex motor response sequence execution to a higher degree than alcohol hangover, which did not differ from the sober state. The fact that this ESD effect was only reflected by the MSD measure suggests that it mainly arose from intoxication-associated automatization deficits during motor response sequence execution. Secondly, we found that unlike sober and hungover participants, intoxicated participants did not display a learning effect. The fact that this ESD effect was only reflected by the RT1 measure suggests that it mainly arose from intoxication-associated learning deficits in response selection and pre-motor planning of the motor response sequence.

## Discussion

Alcohol-related cognitive control deficits have been widely researched, but still, rather little is known about how alcohol affects the establishment of automatisms and the balance between goal-directed and habitual behavior. This is however all the more important as an imbalance towards decreased control and increased habitual actions fosters the development and maintenance of an alcohol use disorder (AUD). In line with this, it has also remained fairly unclear how the different effects of binge drinking (e.g., alcohol intoxication and alcohol hangover) affect the balance between controlled and automated (pre-)motor processes, especially with respect to the latter. Specifically, it has remained unclear how these two alcohol-induced states affect the acquisition of new automatisms, even though a faster/more pronounced acquisition of automatisms may potentially contribute to (relative) control deficits observed during alcohol intoxication and hangover. Therefore, the aim of this study was to investigate acute and post-acute alcohol effects on the controlled vs. automatized planning and execution of complex motor response sequences in healthy young males. For this, we used a mixed study design in which each participant was either assigned to the intoxication group or the hangover group and completed a newly developed paradigm once sober and once at an alcohol appointment. Of note, the motor response sequences in this paradigm reflected sequences of S-R associations, as single stimuli were used to trigger each single motor responses within the motor response sequence.

### Task effects

Regarding the newly developed task itself, we investigated the emergence of automated processes. This was done by comparing situations that require top-down controlled motor response sequence generation to situations where motor response sequence generation could become automatized based on the repeated presentation of the same stimulus array (and thus the requirement to repeatedly perform the same motor response sequence) over the course of the experiment. When motor response sequence automatization was possible, participants could rely on less cognitively effortful, bottom up motor response generation in order to execute the correct motor response sequence. We expected that this automatization should be increasingly established with increasing repetition (automatization effect). In contrast to this, participants had to rely on top-down cognitive control in order to execute the correct response whenever motor response sequence automatization was not possible (i.e., when the required motor response sequence was not predictable). We therefore hypothesized that complex motor response sequence performance should be more accurate and faster in the auto condition (i.e., when automatization was possible) than in the control condition (i.e., when automatization was not possible). We furthermore hypothesized that this performance gap should increase from block 1 to block 5 (i.e., over the course of the experiment) due to an increasing degree of automatization in the auto condition.

These hypotheses were fully confirmed by the interaction between condition and block in the ESD measure. This measure reflects the duration of the entire process, i.e., from stimulus recognition to response selection and then to motor response sequence execution. Specifically, task performance was faster in situations when automatization of the motor response sequence was possible compared to situations when automatization of motor response sequences was not possible. This automatization effect was significantly larger in the last block, as compared to the first block of the experiment. Importantly, this demonstrates that motor response sequence generation was increasingly automated with increasing repetition. We therefore concluded that the newly developed task does in fact allow to investigate the automatization of complex motor response sequences as compared to their top-down execution.

Regarding accuracy, the hypothesized result pattern was only partly obtained: We found more accurate performance when motor response sequence automatization was possible as compared to when it was not possible. However, this automatization effect did not increase over the course of the experiment. As the accuracy was already quite high in the first auto block (around 94%), it is however possible that a potential ceiling effect prevented further significant improvement from there to the last automated block at the end of the task. Irrespective of whether this was indeed the underlying reason for the lack of increasing automatization effects over the course of the experiment, this clearly shows that accuracy does not reflect the predicted automatization of complex motor response sequences as well as the ESD measure.

In conclusion, the ESD showed to be the more sensitive and suitable measure to investigate automatization processes of complex motor response sequences in this study. Therefore, we limited the alcohol-related analyses to the ESD measures and included motor sequence duration (MSD; i.e., the time elapsed between first and last response) and first motor response time (RT1; i.e., the time elapsed between stimulus onset and first motor response) in follow-up analyses to further clarify the origin of the observed ESD effects. This will be detailed in the following sections.

### Alcohol effects

**Figure 4:**
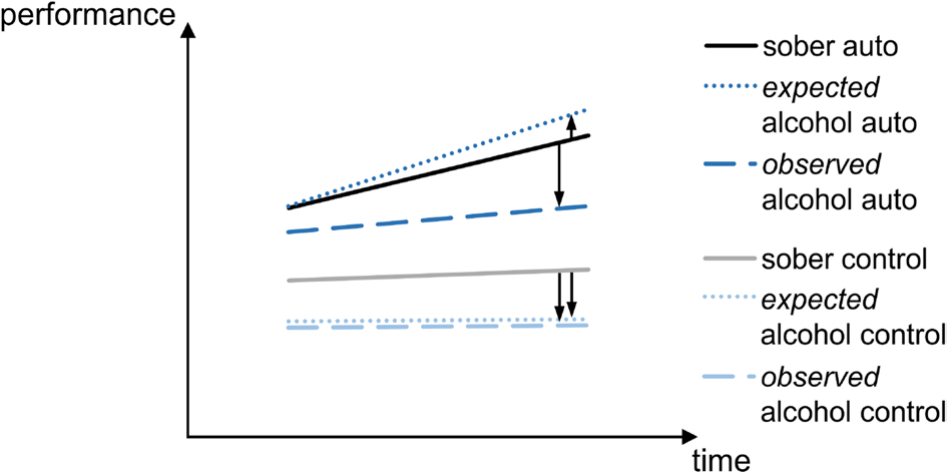
Schematic illustration of expected vs. observed alcohol intoxication effects on automated vs. controlled motor response sequence generation, as compared to sobriety. We had expected and also observed alcohol-related impairments in controlled motor response sequence generation. Furthermore, we had expected the automatization of response sequences to be relatively preserved during intoxication. In contrast to our initial hypothesis, we however observed impaired automatization of motor response sequences during acute intoxication.

Based on previous findings that controlled processes seem to be more impaired by alcohol than automated ones^13,15–17^, we had expected that alcohol should have a (more) detrimental effect on controlled motor response sequence generation and execution, while automatized motor response sequence generation and execution should remain (largely) preserved (see Fig. 4). We had further hypothesized that these effects should be more pronounced towards the end of the experiment, when automated processes should become more consolidated. Additionally, we had expected that acute intoxication should have a greater impact on controlled motor response sequence generation than alcohol hangover. This would have shown in the interaction of all four factors (i.e., alcohol administration, alcohol manipulation group, task condition, and block number). However, we did not find this four-way interaction to be significant. Instead, we observed that alcohol impaired motor response sequences in two different ways, which was shown in two separate three-way interactions. On the one hand, we found alcohol effects on the automatization of motor response execution, and on the other hand, we found alcohol effects on the learning of response selection and pre-motor planning.

#### Alcohol effects on the automated response execution of motor response sequences

Partly opposing our initial hypotheses, we found stronger intoxication-related impairments when motor response sequence automatization was possible as compared to when it was not possible. Alcohol hangover had no impact on automated or controlled processes (as compared to sobriety). The automatization effect itself (i.e., the difference between control and auto condition performance) was still present in both groups and at both appointments (i.e., sober and alcohol), which indicates that the automatization of complex motor response sequences still took place in both alcohol states. In other words, the formation and benefit of new habits/automatisms was still possible under the influence of alcohol, albeit at a generally lower level during acute intoxication. The fact that we found this ESD result pattern to be more likely to be reflected in the MSD measure (than in the RT1 measure) suggests that these intoxication-related impairments mainly arose from deficits in processes related to motor response sequence execution (but not to the processes associated with selecting and planning the required motor responses). That is, intoxication was more detrimental to the automatization of motor response sequence execution than being sober or hungover. In comparison to this, controlled motor response sequence execution seemed to be less affected by alcohol, even though performance was still worse than during the automatization condition.

At first glance, these intoxication effects might seem to contradict previous studies, which mainly reported stronger impairments in controlled behavior, than in automatic behavior^13,15,16^. Yet, differences in the investigated functional domains may provide an explanation when taking a closer look: Other than previously used paradigms like the Simon No-Go task^13^ or the stimulus–response-binding task^15^, the task we used in the current study did not rely on already existing automatisms, like the tendency to respond on the side where a stimulus occurs, or the tendency to automatically associate events and responses that co-occur. Instead, the task used in this study required to form new, explicit automatisms as the task proceeded. It is thus possible that the different findings of intoxication effects on automated processes could be attributed to differently affected functional domains: The explicit acquisition of new habits or automatisms may be impaired by intoxication as shown in our study, while the execution of pre-established and/or partly implicit automatisms may remain relatively stable^13,15^. In line with our results, Obst and colleagues (2018) showed that alcohol impaired habitual decision making in a two-step task, instead of promoting it^57^. They further demonstrated a shift away from habitual behavior and towards goal-directed behavior in intermediate-risk drinkers as compared to low-risk drinkers, which further stresses the fact that there may be certain conditions under which alcohol impairs the habitualization of behavior and instead promotes top-down controlled response selection.

The lack of significant hangover effects is in line with previous findings investigating the potential effect of hangover on the interplay between controlled and automated processes^26^, where participants showed no differential effects on automated vs. controlled processes. Likewise, alcohol hangover did not affect the balance between model-based or model-free behavior^28^. Further evidence on how alcohol hangover affects automated processes or motor response sequence execution is however still rather rare.

In summary, alcohol hangover did not differentially affect controlled vs. automated processes, while acute intoxication likely impaired the automatization of complex motor response sequence execution to a stronger degree than the top-down execution of motor response sequences. In this context, it should however be noted that while automatization effects were reduced in size, they were still evident during acute intoxication.

#### Alcohol effects on response selection and planning-related learning

Interestingly, we furthermore found intoxication-induced learning impairments that seemed to be independent from the aforementioned automatization deficits. Specifically, acutely intoxicated participants failed to show any learning effects (i.e., behavioral performance improvements from block 1 to block 5). While we had expected an intoxication-related reduction in the learning effect, this did however not interact with automatization, thus suggesting that the effects of acute alcohol intoxication onto learning and automatization might not be mediated via the same mechanisms. The fact that this ESD result pattern was reflected in the RT1 measure (but not in the MSD measure) suggests that the intoxication-induced learning deficits most likely arose from impairments in response selection and planning (but not in motor response sequence execution). That is, acute intoxication was detrimental to the learning effect arising from the repeated planning of complex motor response sequences, while alcohol hangover did not affect these learning processes. This finding is partly supported by a study that reported impaired motor preparation of 3-key responses in hazardous drinkers as compared to healthy controls^58^. Importantly, both these results as well as our observations in healthy young participants demonstrated alcohol-related learning deficits in pre-motor planning. Taken together, this suggests that alcohol is a causal factor for deficits in planning complex motor response sequences. Yet, motor response planning and associated learning effects can take place both explicitly and implicitly. In the task used for this study, learning took place on an explicit level, as the same geometric figure configuration was presented in 70% of the auto condition trials. Previous studies suggested that acute alcohol intoxication selectively impairs explicit learning processes^59–61^, as implicit learning was not equally impaired during alcohol intoxication^59,60,62,63^. Likewise, implicit sequence learning did neither differ between abstinent alcohol-dependent patients and healthy controls^64^, nor in Korsakoff’s syndrome patients^65^. These findings suggest that alcohol may have a dissociative effect on learning: While alcohol intoxication seems to impair explicit learning, implicit learning might remain largely stable. Importantly, this implies that the type of learning might be a major determinant in whether or not detrimental effects of intoxication can be observed (please refer to the “Outlook” section for further implications).

Alcohol hangover had no impact on the general learning effect as compared to sobriety. The lack of hangover effects on response selection and planning is (partly) in line with a previous publication of our group demonstrating that hungover participants showed no significant decline in response selection, even though Bayesian analysis provided evidence for slightly decreased response selection in hungover participants, as compared to sober participants^26^. Furthermore, alcohol hangover had no effect on explicit learning as measured by word-learning tasks^4,7,66^, although some other studies found significant hangover-induced learning impairments^29,67^. As suggested before, more research on how alcohol hangover affects motor response sequence learning is needed.

In summary, alcohol hangover did not modulate explicit motor response learning effects, but acute intoxication eradicated any learning effects based on the repetition of response selection and planning. Importantly, this effect seemed to be independent of the observed alcohol effects onto automatization, which were not only reflected by a different interaction, but also in a different behavioral measure.

### Limitations

Although our newly developed paradigm allows investigating how complex motor response sequence automatisms are established (as it is very likely that the sequence trial will be holistically processed with increasing automatization), it does not allow investigating implicit learning, implicitly established automatisms, or pre-established/inherent automatisms. Given the potential distinction between implicit and explicit learning outlined in the previous text section, more research is needed on these issues, specifically when considering that AUD patients tend to involuntarily shift away from goal-directed towards habitual behavior.

We refrained from using a placebo condition because initial piloting had shown that the difference between a placebo and the large amounts of alcohol administered by us was very obvious, as all pilot participants could easily identify the placebo. Knowledge about the intoxication and hangover investigated in this study might therefore have produced expectancy effects and/or compensatory effort during task performance. While this is unfortunately largely unavoidable in case of high-dose intoxication, Devenney et al.^68^ demonstrated that this does not necessarily apply in case of alcohol hangover, as they found no expectancy effects on the cognitive performance in the hangover state when informing one of their study groups about the study purpose.

Further limitations include gender and age. Based on the decision of the local ethics committee, females were not allowed to partake in this study. This is rather unfortunate because previous findings indicated that compared to males, females may be more prone to hangover^69^ and deficits in cognitive performance after heavy alcohol consumption^70–72^, although this effect was not unequivocally reported by all studies^73^. Females have a lower first-pass metabolism due to lower ADH enzyme activity^74,75^, so that alcohol persists for longer in their system^76^. For these reasons, it is conceivable that females may show stronger cognitive impairments during acute intoxication and/or alcohol hangover than males. Lastly, we did not recruit individuals older than 30 years as we intended to obtain a homogeneous and healthy sample. However, the occurrence of alcohol-related hangover and the severity of hangover symptoms seem to change with age^77,78^. In order to obtain a more complete picture of the acute and post-acute effects of alcohol consumption, it would be therefore appropriate to include both females and adults of middle to older age in future studies.

### Outlook

In this study, we used a paradigm for which a new automatization of motor response sequences was established in an explicit way via the repetition of the same stimulus (and response) combination. On a neurobiological level, NMDA glutamate receptor activation has been shown to play an important role in (explicit) learning^79,80^. This might explain some of our findings as acute ethanol intoxication inhibits NMDA receptor activity^81–83^, which may in turn have hampered explicit learning. Given that implicit learning has so far not been shown to be equally impaired by alcohol intoxication^59,60,62,63^, it is hence possible that we observed intoxication-induced deficits in automatization and learning effects due to the explicit nature of the task. Because this does however not necessarily have to be the case in an implicit learning task, this should motivate further studies investigating whether and how implicitly acquired motor response sequence automatisms are affected by acute intoxication and alcohol hangover. As the selection and execution of pre-existing automatisms also seem to be rather unaffected by alcohol^13,17,19^, it could further be speculated that AUD patients and/or binge-drinkers strongly rely on habits because the necessary automatisms have already been previously established (inherently or potentially through implicit learning), and not because the explicit acquisition of new habits is facilitated during intoxication. Yet, this remains to be investigated in future studies.

Concerning the differential effects of acute intoxication and alcohol hangover, a possible explanation might be found in the opposing effects of ethanol and its major metabolite acetaldehyde on GABAergic neurotransmission^84^. In fact, it has been shown that GABA plays an important role in motor learning^85,86^. Accordingly, stronger GABA-mediated inhibition was associated with poorer performance in a motor sequence learning task^85^. As acute ethanol intoxication enhances GABAergic signaling^87^, this mechanism may have led to the intoxication-impaired motor response sequences learning in our study as well. In contrast to this, there might be no comparable motor learning deficits during alcohol hangover, as acetaldehyde seems to reduce the activity of the GABAergic system^84,88^. These issues also deserve future investigations.

## Conclusion

To sum up, we investigated whether alcohol leads to a stronger impairment of top-down controlled motor response sequences, as compared to the establishment and execution of motor response sequence automatisms. Interestingly, we did not observe an alcohol-induced shift from controlled to habitual behavior, which would have indicated a shift in the “metacontrol” of the balance between those two functionally opposing operation modes. Instead, we observed two statistically independent effects reflecting that alcohol intoxication (but not hangover) impaired two different processes of complex motor response sequences. Partly opposing our initial hypothesis, we found that alcohol intoxication interfered with the automatization of motor response sequences more strongly than with their controlled execution. Moreover, alcohol intoxication eradicated learning effects on response selection and planning, irrespective of automatization. The finding that the planning of motor response sequences as well as the automatization of motor response sequence execution are both impaired by intoxication speaks against the hypothesis that automatization tendencies are generally and unequivocally spared and/or reinforced by acute intoxication. While our findings contradict the assumption that alcohol intoxication facilitates the acquisition of explicit learning and automatization, this does however not exclude the possibility that acute intoxication may improve the execution of pre-existing automatisms and/or the implicit acquisition of motor response sequence automatisms.

## Methods

### Participants

Given that we used a newly developed paradigm, we did an a priori estimation of the required sample size for our study design using G*power software^89^. Yielding for a small-to-medium effect size of *f* = 0.15^90^ with an alpha error probability of 5%, a power of 95%, two alcohol manipulation groups (intoxication vs. hangover), eight measurements (appointment [sober vs. alcohol] x task condition [control vs. auto] x task block [block 1 vs. block 5]), and an assumed correlation among repeated measures of *r* = 0.5, we obtained a sample size of *n* = 62. To compensate for potential issues and dropouts, we initially recruited *n* = 76 healthy males aged between 18 and 30 years for this study. All participants had normal or corrected-to-normal vision, no prior history of physical, neurological or mental illness, and no acute or chronic medication intake that could influence liver or kidney function, or the central nervous system. The participants’ eligibility was determined via a telephone screening. This included the assessment of their physical and mental health with a semi-structured interview and their drinking pattern using the alcohol use disorders identification test (AUDIT)^91^. Individuals who reported alcohol-induced failure to perform daily routines and/or alcohol-related memory problems and/or binge drinking (i.e., consuming eight or more standard units of alcohol per occasion) “daily” or “almost daily”, were excluded because this would have indicated extensive problems associated with alcohol (ab)use. In order to minimize the risk of intoxication-associated complications, we furthermore required that all participants had practiced voluntary binge drinking at least once within the past 12 months. Further inclusion criteria on drinking patterns slightly varied between the intoxication and the hangover group, as the administered amount of alcohol was lower in the intoxication group than in the hangover group (please refer to the “Experimental procedure and alcohol administration” section). The reason for this was that we were required to only include participants who reported to have recently (within the last year) tolerated alcohol amounts comparable to those administered in their experimental group, and the absolute amount administered in the hangover group was higher than that administered in the intoxication group—even though we had of course planned to achieve comparable maximal intoxication levels in both experimental groups.

For the intoxication group, individuals who reported to binge drink at least once a year but no more than once a month, and who had an overall moderate/ non-high AUD risk pattern of alcohol use were included. This was defined by AUDIT scores between 1 and 15 points^91^. For the hangover group, individuals who reported to binge drink at least once a month but no more than weekly/less than (almost) daily, and who had an overall moderate to harmful pattern of alcohol use, but without AUD diagnosis according to the ICD-10 criteria, were included. This was defined by AUDIT scores between 2 and 19 points^91^. Experienced psychologists screened all participants to make sure that none of them met the full diagnostic criteria required for an AUD diagnosis according to ICD-10. All participants provided written informed consent prior the beginning of the experiment and received a financial reimbursement (intoxication group: 70 € in total, hangover group: 80 € in total) or study credits. The study was conducted in accordance with the Declaration of Helsinki and approved by the Ethics Committee of the Faculty of Medicine of the TU Dresden (ID number: EK293082014).

### Experimental design

**Figure 5:**
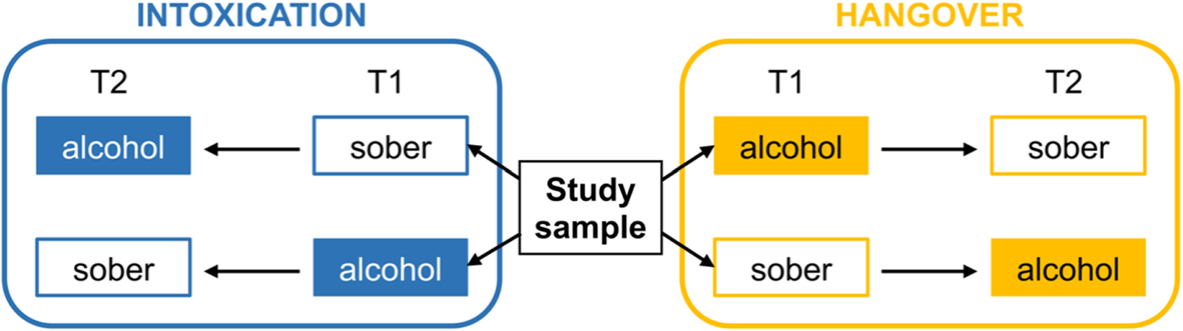
Schematic study design. The between-subject factors were alcohol manipulation group (intoxication vs. hangover) and appointment order (alcohol on first (T1) vs. second (T2) appointment). The within-subject factor was alcohol administration (sober vs. alcohol appointment).

In this study, we used a mixed 2 × 2 × 2 experimental design, in which the within-subject factor alcohol administration (alcohol vs. sober), and the between-subject factors appointment order (alcohol on first vs. second appointment) and alcohol manipulation group (intoxicated vs. hungover) were systematically varied. This is illustrated in Fig. 5.

Each participant was tested twice, i.e., once sober and once under the influence of alcohol. Regarding the alcohol manipulation, each participant was either assigned to the intoxication group or to the hangover group. The appointment order was balanced across all participants so that half of the participants had their sober appointment before their alcohol appointment, whereas the other half had their alcohol appointment before their sober appointment. For this reason, systematic differences between the first and second appointment could not be investigated in the statistical analyses reported in the main text, but we provided additional analyses on the order irrespective of the alcohol manipulation (T1 vs. T2) in the Supplementary Material. Both appointments were scheduled for a minimum of 48 hours and a maximum of 7 days apart from each other. All participants were asked to refrain from the use of caffeine, guanine, nicotine and all other sedative or stimulant substances within the last four hours prior to the start of each appointment. At the beginning of each appointment and at several time points during acute intoxication, breath alcohol concentration (BrAC) was assessed using the breathalyzer “Alcotest 3000” (Drägerwerk, Lübeck, Germany). Participants were required to be sober (BrAC = 0.00 ‰) at the beginning of each appointment. The experimental procedure only resumed when sobriety was given.

### Experimental procedure and alcohol administration

**Figure 6:**
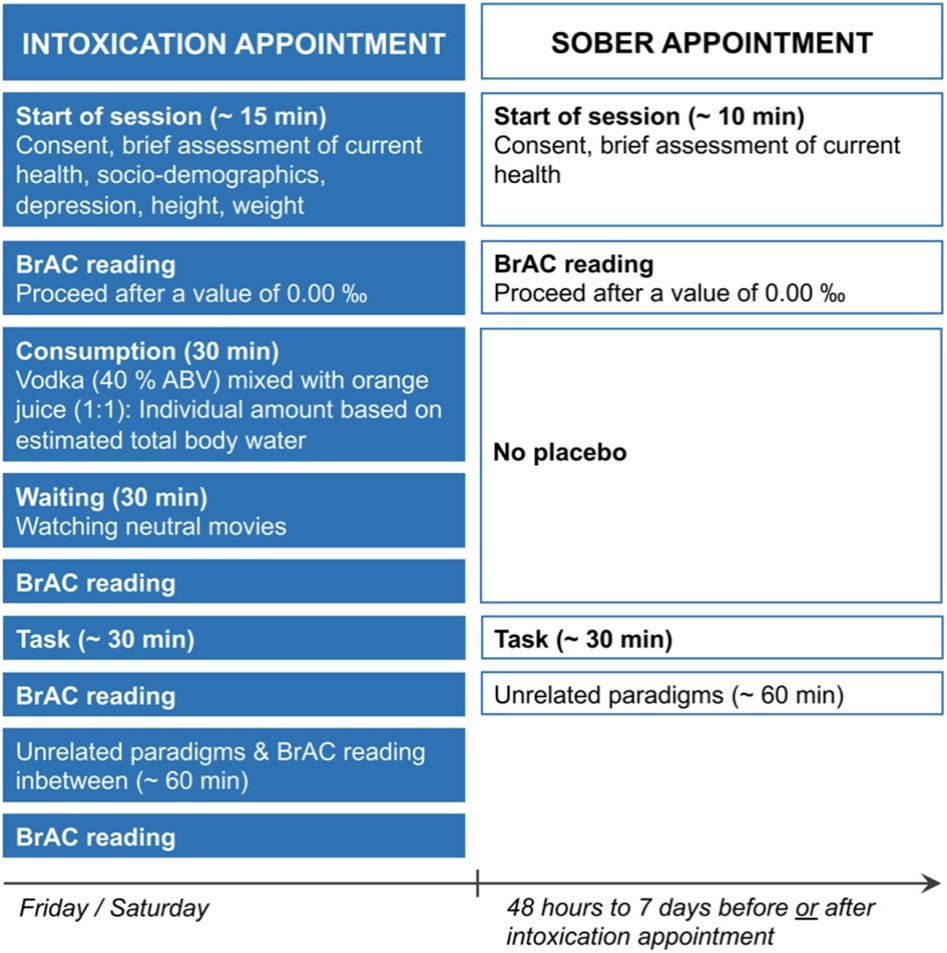
Visualization of the experimental procedure in the intoxication group. Each participant was tested once intoxicated and once sober. Appointment order was balanced across the group.

**Figure 7:**
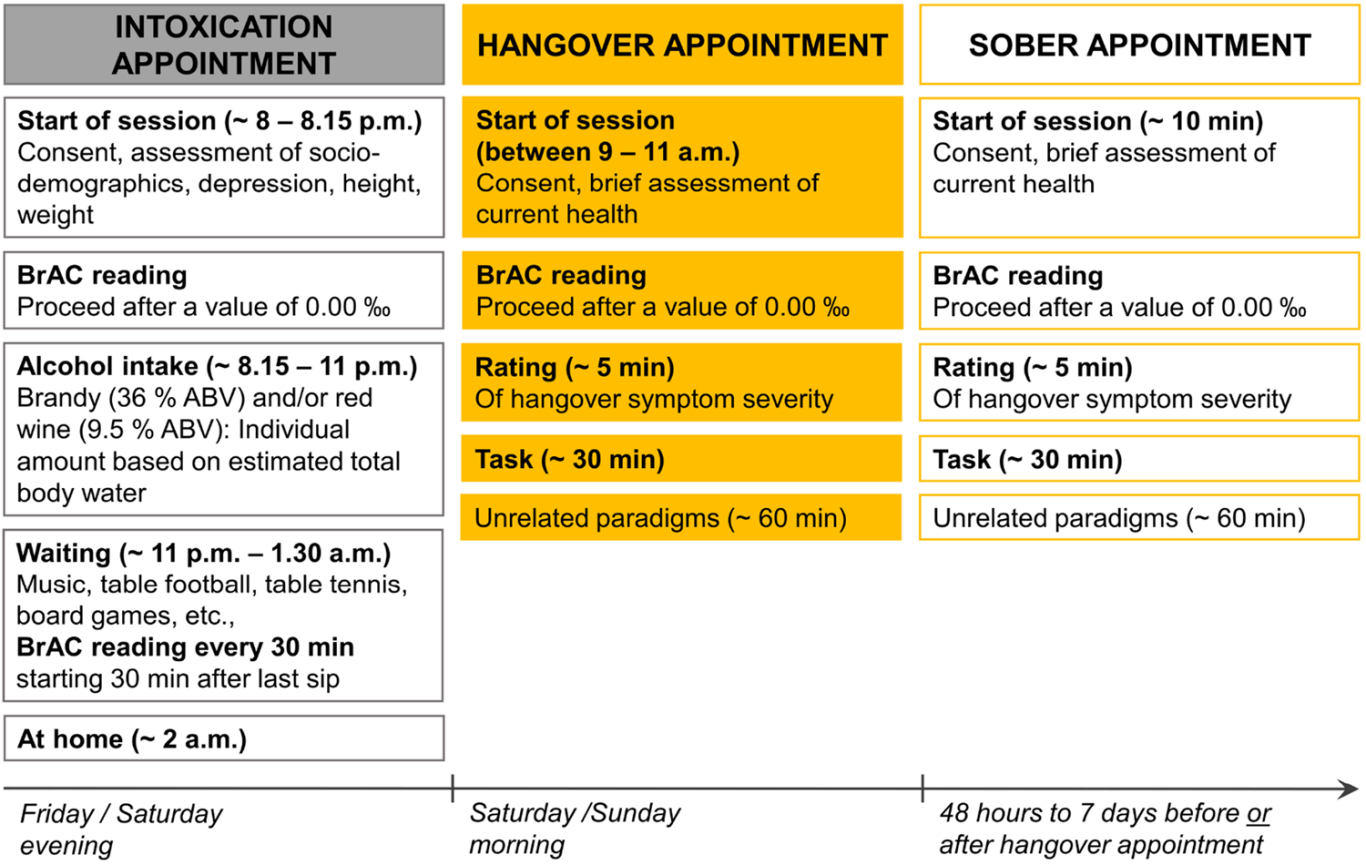
Visualization of the experimental procedure in the hangover group. Each participant was tested once hungover after a night of being experimentally intoxicated at our facilities and once sober. Appointment order was balanced across the group.

For each alcohol manipulation group, the experimental procedure and the alcohol administration followed protocols that had already been used in previous studies on alcohol intoxication or alcohol hangover, respectively^12,15,26,92^. An overview of the study protocols is provided in Figs. 6 and 7, while the details of those protocols are provided in the Supplementary Material. All participants were experimentally intoxicated in a controlled laboratory environment. Participants in the intoxication group were administered 1.98 g of alcohol per liter of estimated total body water (TBW) on an empty stomach within 30 minutes, while participants in the hangover group were administered 2.64 g of alcohol per liter of estimated TBW on a full stomach within 2 hours or more. Based on the assumption of different resorption deficits on an empty vs. full stomach (20% vs. 30–40%), we however aimed for average maximal BrACs of approximately 1.2 ‰ in both experimental groups.

### Questionnaires

At the beginning of their respective intoxication appointment (and thus before alcohol administration), all participants provided information on height, weight, and socio-demographic characteristics and filled in the Beck Depression Inventory^93,94^ to determine potential depressive symptoms. In the hangover group, participants additionally rated the severity of 24 hangover symptoms on an 11-point Likert scale, which reached from 0 (non-existent) to 10 (extreme), at the beginning of both their sober and their hangover appointment. For this purpose, we used the hangover symptoms list introduced by Hogewoning and colleagues^95^ and added another item to asses sleep problems.

### Task

**Figure 8:**
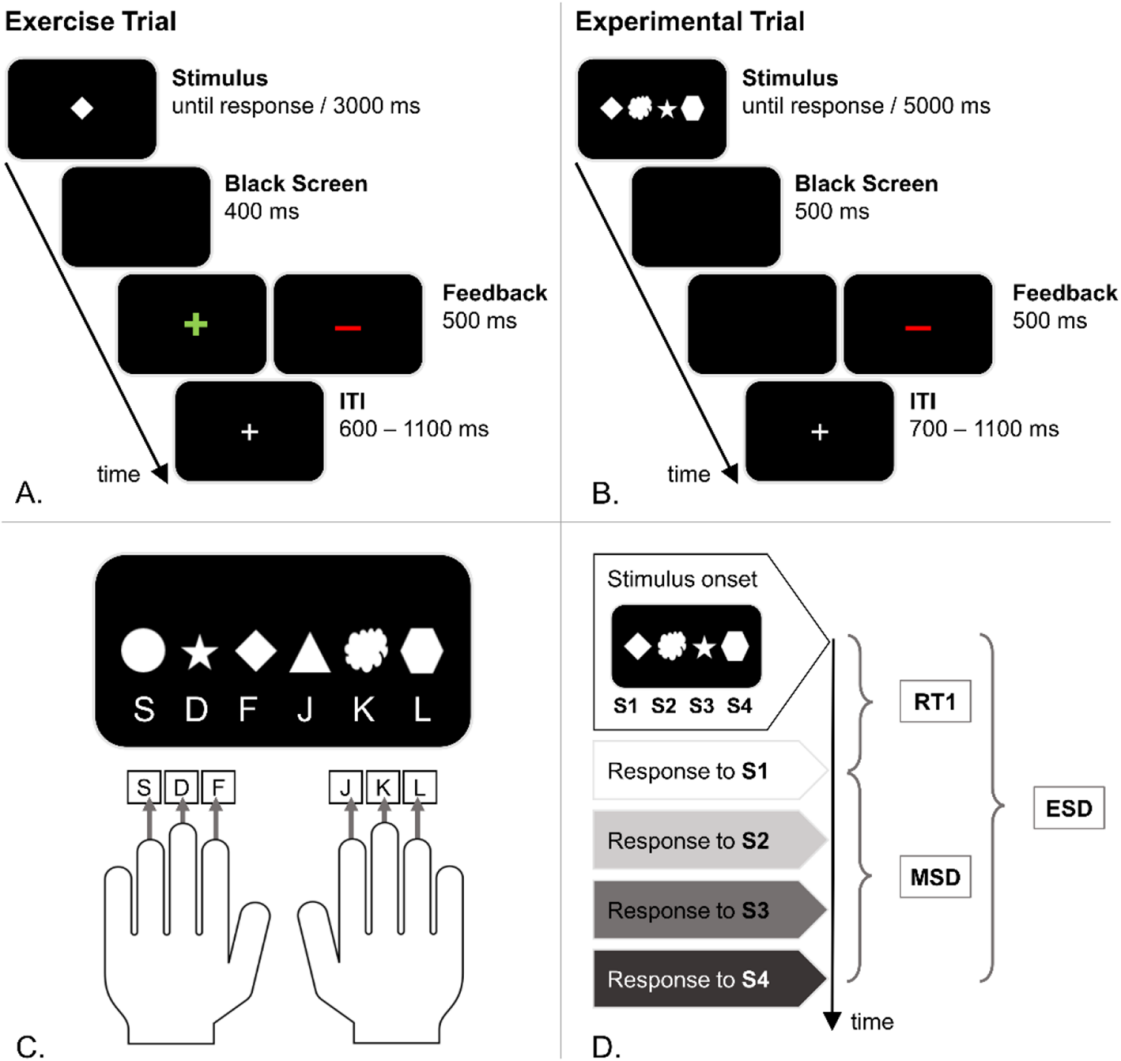
Outline of the newly developed habit paradigm. (**A**) Illustration of the time course of an exemplary single trial in milliseconds (ms) during the exercise. Feedback was given in each trial (green plus sign after correct responses; red minus sign in case of a wrong or missed response). The inter-trial interval (ITI) was jittered. (**B**) Illustration of the time course of a single trial in milliseconds (ms) during the experiment. Negative feedback (red minus) was given in case of a wrong or missed response. No feedback (i.e., a black screen) was displayed in case all four responses were correct. The inter-trial interval (ITI) was jittered. (**C**) Overview of task-relevant stimulus–response (S-R) associations, which were established during the exercise. (**D**) Overview of the obtained response time measures. The first motor response (RT1) defines the time passed between stimulus onset and the first response. The motor sequence duration (MSD) is defined as the time passed between first and last response. The entire sequence duration (ESD) is defined as the time passed between stimulus onset and the last response.

We used a newly developed experimental paradigm to investigate the automatization of motor response sequences. This was done by comparing the execution of complex motor response sequences in situations that require top-down controlled motor response sequence generation to situations where motor response sequence generation could be automatized. Specifically, the motor response sequences generated in this paradigm were sequences of S-R associations, as single stimuli triggered single motor responses within the motor response sequence. The task is visualized in Fig. 8.

The task was presented on a 17'' high quality flat screen monitor with a refresh rate of 144 Hz in a well-lit and quiet room. Stimulus presentation and recording of the behavioral responses were implemented using Presentation® software (Version 16.5, Neurobehavioral Systems, Inc., Berkeley, CA, USA). The task comprised an exercise and the actual experiment.

#### Exercise

The aim of the exercise was to establish single S-R associations between visual stimuli and response keys. Importantly, this pre-experimental establishment of single S-R associations allows to investigate the controlled vs. automatized generation of motor response sequences free of potentially (strong) confounding effects stemming from inter-individual differences in the establishment of single S-R associations. For this reason, the main exercise was always conducted while the participants were still sober. As this created a temporal gap of about 60 min between exercise and experiment when testing acute intoxication effects (but not at the sober appointment, or in the hangover group), participants received an additional brief “fresh up” exercise right before starting their task performance while intoxicated. A total of six simple geometric figures were linked to six different keys on a standard QWERTZ keyboard. This is detailed in Fig. 8C. In the exercise, participants were instructed to respond to single geometric figures by pressing the corresponding key with the respective finger to make sure all participants had formed stable S-R associations for all stimuli and responses before starting the actual experiment. Participants were asked to keep their fingers on the response keys during the entire duration of the exercise. Each trial (compare Fig. 8A) started with the central presentation of one of the six white geometric figures on black background. Stimulus presentation ended with a key press or after 3000 ms (in case of a missed response). A black screen was then displayed for 400–500 ms, after which feedback was given. In case of a wrong or missed response, a red minus sign was centrally presented for 500 ms at T1 and 1000 ms at T2. In case of a correct response, a green plus sign was centrally presented for 500 ms at T1 and 1000 ms at T2. Eventually, a white fixation cross was presented during the inter-trial interval, which randomly varied between 600 and 1100 ms. On the first appointment, the exercise consisted of 20 mandatory blocks. The first ten blocks comprised six trials each. In each of these blocks, every single geometric figure was presented once in a fixed order (starting with the respective key press from left to right on the keyboard). The subsequent ten blocks comprised twelve trials each. In each of those blocks, every single figure was presented twice in a randomized order. The exercise at T1 took approximately 10–15 min. On the second appointment, each block contained twelve trials, that is, every single figure was randomly presented twice. The exercise was finished as soon as one whole block (i.e., 12 trials in succession) was performed without any errors or misses, which took roughly 2–5 min. Due to the brief interval between both appointments, a short task refreshing of the pre-established S-R associations on the second appointment was sufficient to reactivate the single stimulus S-R associations at T2. In case either the experimenters or the participants did not feel that stable S-R associations had been formed during the exercise, the exercise was repeated until that point was reached.

#### Experiment

In the actual experiment, participants were instructed to respond to four geometric figures by successively pressing the corresponding keys as quickly and as accurately as possible when “reading” them from left to right. In each trial (compare Fig. 8B), an array consisting of four horizontally aligned white geometric figures was centrally presented on black background. Stimulus presentation ended with the fourth key press or after 5000 ms. After stimulus offset, a black screen was displayed for 500 ms. In case of at least one wrong or missed response, feedback was given by a red minus centrally presented for 500 ms. In case all four responses were correct, another black screen was displayed for 500 ms. Eventually, a white fixation cross was presented during the inter-trial interval, which randomly varied between 700 and 1100 ms. The experiment comprised 300 trials, which were divided into ten blocks of equal size (i.e., 30 trials each). Out of these, five blocks were so-called “control” blocks and the other five blocks were “auto(matization)” blocks. The experiment always started with a control block and the two block types alternated, so that all odd blocks were control blocks and all even-numbered blocks were auto blocks. In control blocks, the occurrence and configuration of the four geometric figures were randomized in each trial. Because the figure configuration was not predictable in any of the control block trials, participants had to rely on top-down cognitive control in order to configure and execute the correct motor response sequence. In auto blocks, the configuration of the four geometric figures was identical in 70% of all trials (auto identical trials; i.e., the same four figures were presented in the same order, thus requiring the same motor response sequence), while the figure configuration was randomized in the remaining 30% of all trials (comparable to the control block trials). While the randomized trials prevented premature responding before/upon stimulus onset, the frequent repetition of the same figure configuration in the auto identical trials allowed for the automatization of motor response sequence generation. This means that in auto identical trials, participants could rely on less cognitively effortful, bottom up motor response generation in order to execute the right response. With increasing repetitions (i.e., with increasing block numbers), this automatization is increasingly established. This should be reflected in faster and more accurate responses in auto identical trials, as compared to control trials. Importantly, the figure configuration of the auto identical trials differed between first and second appointment (at the first appointment: circle, cloud, hexagon, diamond; at the second appointment: triangle, star, circle, hexagon), but always required two responses from each hand. This was done to prevent/minimize learning effects between the two study appointments. The experiment took approximately 25 min and participants were offered to take breaks after each block.

Eventually, the block conditions control and auto, of which only auto identical trials were kept, and the respectively first and last (i.e., fifth) block entered the statistical analyses. Behavioral measures that can be obtained from the task (please see Fig. 8D) are accuracy (i.e., percentage of trials with four correct responses) and entire sequence duration (ESD), which can be further subdivided into first motor response time (RT1) and motor sequence duration (MSD). ESD is the total response time from stimulus onset to the fourth response and thus reflects overall differences in automatic vs. controlled motor response sequence generation that modulate the duration of this process. RT1 is the time between stimulus onset and the first response, thus mainly reflecting cognitive processes involved in planning and selection of the motor response sequence. MSD is the duration of the motor response sequence itself (i.e., the time from the first to the fourth response), thus mainly reflecting processes involved in the coordination and execution of the motor response sequence.

### Statistical analyses

Only trials in which participants responded correctly to all four geometric figures (within a time window of 200–3000 ms after stimulus onset for the first response and within a time window of 25–1500 ms after each previous key press for the second to fourth response) entered statistical analyses. In case this exclusion procedure resulted in less than 10 remaining trials in any of the condition combinations (i.e., in any block), the participant was excluded from the analyses.

The obtained accuracy and ESD measures were analyzed using separate mixed ANOVAs with the between-subject factor alcohol manipulation group (intoxicated vs. hungover), and the three within-subject factors alcohol administration (sober vs. alcohol), condition (control vs. automatization), and block (block 1 vs. block 5). To determine the most likely source of the obtained ESD effects, we subsequently ran the same mixed ANOVA separately for RT1 and for MSD. The degrees of freedom were Greenhouse–Geisser corrected whenever necessary. Behavioral variables were tested for variance homogeneity using Levene’s tests and for normal distribution using Kolmogorov–Smirnov tests. If these assumptions were violated, Welch-tests (in case of variance heterogeneity) and Mann–Whitney *U* tests or Wilcoxon signed-rank tests (in case of deviation from normality) were used to retest the significance of main effects and post hoc comparisons. The mean, minimum and maximum value, as well as the standard error of the mean as a measure of variability were reported for the presentation of descriptive statistics.

## Supplementary Information

﻿Supplementary Information 1.

Supplementary Information 2.

## Data Availability

The datasets generated during and/or analyzed during the current study is available online via https://osf.io/jmxac/.
