# Supplementary material for: Alcohol intoxication, but not hangover, differentially impairs learning and automatization of complex motor response sequences
Source: Sci Rep. 2021 Jun 15;11:12539. doi: 10.1038/s41598-021-90803-5 (PMC8206163; doi:10.1038/s41598-021-90803-5)
Supplement: Supplementary file 2 — Supplementary Information 2. [file 41598_2021_90803_MOESM2_ESM.pdf]

Supplementary Information

Article title: Alcohol intoxication, but not hangover, differentially impairs learning and automatization of complex motor response sequences
Journal name: Scientific Reports
Authors: Opitz A, Ghin F, Hubert J, Verster JC, Beste C, Stock AK
Corresponding author: Antje.Opitz@ukdd.de

Results

Graphic overview of alcohol effects on each behavioral measure and experimental group

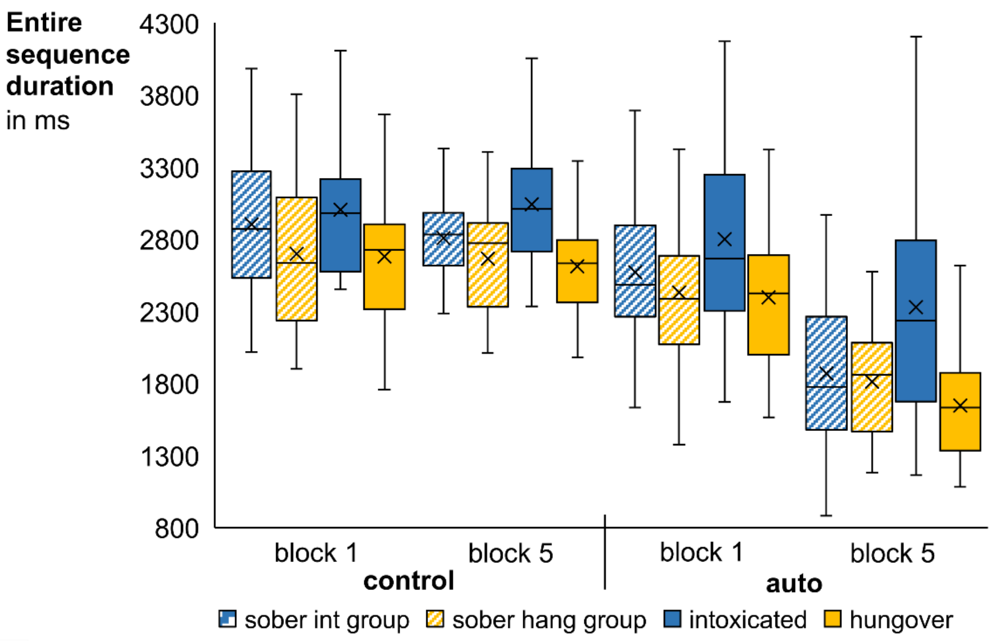

Figure S1. Box plots of the entire sequence duration (ESD) for each combination of task condition, block, alcohol manipulation group, and alcohol administration. We observed significantly longer ESDs in the intoxication group than in the hangover group. Besides a significant interaction between alcohol administration and alcohol manipulation group, we observed two three-way interactions, that is between alcohol administration x condition x alcohol manipulation group, and alcohol administration x block x alcohol manipulation group. Figure 3 and 4 in the main manuscript provide further insights on these three-way interactions.

Hangover symptoms within the hangover group

The rating of most hangover symptoms, including overall hangover severity, were significantly higher during the hungover appointment than during the sober appointment (see **Table S1**). We refrained from simple corrections (like Bonferroni), as we only wanted to demonstrate the success of hangover induction. And as the single item ratings on the hungover appointment were mostly positively correlated (mean inter-item correlation r = .387; range from -.106 to .828), simple approaches of correcting for multiplicity would have led to a lower power for the detection of symptom differences between appointments.

Table S1. Subjective ratings of sleep duration and hangover symptoms on sober and hangover appointment in the hangover group.

Symptom	Sober	Hangover	$p_{Difference}$
Hours of sleep in previous night	7.40 ± 0.15 (5.5–9)	5.49 ± 0.18 (4–8)	< .001 **
Overall hangover severity	0 ± 0 (0–0)	3.75 ± 0.40 (0–10)	< .001 **
Thirst	0.97 ± 0.28 (0–7)	3.47 ± 0.36 (0–8)	< .001 **
Concentration problems	0.59 ± 0.18 (0–4)	3.22 ± 0.38 (0–8)	< .001 **
Tired	1.15 ± 0.21 (0–4)	4.67 ± 0.39 (1–10)	< .001 **
Sleepiness	1.03 ± 0.23 (0–4)	3.83 ± 0.42 (0–9)	< .001 **
Weakness	0.30 ± 0.11 (0–2)	2.44 ± 0.36 (0–10)	< .001 **
Headache	0.06 ± 0.04 (0–1)	2.64 ± 0.38 (0–8)	< .001 **
Clumsy	0.41 ± 0.15 (0–3)	2.10 ± 0.32 (0–6)	< .001 **
Dizziness	0.03 ± 0.03 (0–1)	1.86 ± 0.32 (0–8)	< .001 **
Sensitivity to light	0.29 ± 0.13 (0–3)	1.61 ± 0.31 (0–8)	< .001 **
Reduced appetite	0.21 ± 0.16 (0–5)	2.11 ± 0.42 (0–9)	.001 **
Sweating	1.24 ± 0.32 (0–7)	1.31 ± 0.30 (0–9)	.670
Nausea	0.03 ± 0.03 (0–1)	1.61 ± 0.37 (0–7)	< .001 **
Apathy	0 ± 0 (0–0)	1.00 ± 0.22 (0–5)	< .001 **
Shivering	0.38 ± 0.13 (0–3)	1.22 ± 0.28 (0–6)	.009 **
Confusion	0.15 ± 0.08 (0–2)	1.08 ± 0.27 (0–7)	.001 **
Heart pounding	0.30 ± 0.10 (0–2)	1.00 ± 0.23 (0–5)	.008 **
Stomach pain	0.24 ± 0.15 (0–5)	1.25 ± 0.36 (0–8)	.006 **
Anxiety	0.47 ± 0.15 (0–3)	1.17 ± 0.33 (0–9)	.015 *
Regret	0.12 ± 0.12 (0–4)	0.97 ± 0.34 (0–10)	.001 **
Sleeping problems	0.21 ± 0.13 (0–4)	1.08 ± 0.35 (0–8)	.015 *
Depression	0.21 ± 0.16 (0–5)	0.72 ± 0.28 (0–9)	.011 *
Vomiting	0.03 ± 0.03 (0–1)	0.78 ± 0.32 (0–9)	.011 *
Heart racing	0.15 ± 0.08 (0–2)	0.64 ± 0.25 (0–6)	.089

Hangover symptoms were rated on an 11-point Likert scale ranging from 0 points (no symptoms) to 10 points (extreme symptoms). Participants were asked to truthfully rate the severity of each symptom on both appointments, irrespective of whether they had been drinking the night before the sober appointment and/or whether they attributed that symptom to alcohol use. In case the average ratings of both appointments were above zero, they were compared with Mann-Whitney U-tests (due to violation of the normal distribution of all sober ratings and most hangover ratings). As all participants rated the symptoms overall hangover severity and apathy with zero points on the sober appointment, we used Wilcoxon-signed rank tests (due to violation of the normal distribution in both cases) to compare the hangover appointment rating against zero. Uncorrected *p*-values are reported in the right column. All values are given as means ± standard error of the mean and the range is given in parentheses. * $p < 0.05$, ** $p < 0.01$.

Order effects: Comparing the first and second appointment

To analyse potential order/learning effects from the first to the second appointment, we ran separate add-on repeated-measures ANOVAs for accuracy and ESD measures using the within-subject factors appointment (first appointment vs. second appointment), condition (control vs. automatization) and block (block 1 vs. block 5). As the order of alcohol administration (sober vs. alcohol) was balanced across the participants of both groups (half of the participants had their sober appointment before their alcohol appointment and vice versa), alcohol administration could not be used as an additional within-subject factor in these analyses. For the same reason, we refrained from including alcohol manipulation group as a between-subject factor. In order to avoid redundancy with the task effects already reported in the main manuscript, we only report the main and interaction effects of the appointment factor below.

Regarding accuracy, there was a main effect of appointment ($F_{(1,69)} = 7.176$; $p = .009$; $\eta^2_p = .094$), with better performance at the second appointment (93.0 % ± 0.6) than at the first appointment (91.0 % ± 0.7). In addition, the interaction between appointment and condition reached significance ($F_{(1,69)} = 9.655$; $p = .003$; $\eta^2_p = .123$). Separate post hoc comparisons showed that participants improved from the first (88.1 % ± 0.8) to the second appointment (91.6 % ± 0.7) in the control condition ($Z = -3.348$; $p < .001$), but not in the automatization condition ($Z = -.888$; $p = .375$). This is however not surprising as the motor sequence required in the auto

identical trials was different between the first and the second appointment in order to avoid aberrant learning effects in automatization. The control condition, however, could not be varied between the first and second appointment due to randomized stimulus generation (please refer to the methods section for details). So while participants learned to respond to a new geometric figure configuration in the automatization blocks at their second appointment, the random geometric figure configurations in the control blocks were already familiar, as they were identical to those of the first appointment. None of the other interaction effects including the appointment factor reached significance (all $F \leq 3.552$; $p \geq .064$).

Regarding ESD, we observed a main effect of appointment ($F_{(1,69)} = 57.843$; $p < .001$; $\eta^2_p = .456$), with faster responses at the second appointment (2369 ms ± 50) than at the first appointment (2649 ms ± 52). There was also an interaction between appointment and condition ($F_{(1,69)} = 22.917$; $p < .001$; $\eta^2_p = .249$). Separate post hoc comparisons revealed that participants responded significantly faster at their second appointment in both the control condition ($Z = -7.102$; $p < .001$) and the automatization condition ($Z = -3.140$; $p = .002$), but the learning effect (first minus second appointment) was significantly greater in the control condition (387 ms ± 30) than in the automatization condition (173 ms ± 53) ($Z = -4.275$; $p < .001$). Like the corresponding interaction effect for the accuracy measure, the greater learning effect in the control condition can be explained with the random, but already known, motor response sequences in the control condition at the second appointment, while a new motor response sequence had to be learned in the automatization condition. Eventually, the interaction between appointment and block also reached significance ($F_{(1,69)} = 21.001$; $p < .001$; $\eta^2_p = .233$). Separate post hoc comparisons showed that participants responded significantly faster at their second appointment both in block 1 ($t_{(69)} = 9.261$; $p < .001$) and in block 5 ($Z = -3.807$; $p < .001$), but the learning effect (first minus second appointment) was significantly greater in block 1 (370 ms ± 40) than in block 5 (189 ms ± 43) ($t_{(69)} = 4.583$; $p < .001$). This effect is also reasonable, because on their first appointment, participants could not benefit from any learning effects in block 1 whatsoever, while there was at least some degree of learning associated with block 5 at both appointments. When comparing the learning effect from first to second appointment in block 1 vs. block 5, we therefore need to keep in mind that participants already processed the respective task blocks on different learning levels. Lastly, the highest interaction effect including the appointment factor did not reach significance ($F_{(1,69)} = 1.797$; $p = .184$).

Methods

Experimental procedure and alcohol administration (detailed)

All participants were experimentally intoxicated in a controlled laboratory environment. For this purpose, we administered individual amounts of alcohol, which were calculated for each participant according to their estimated total body water (TBW). This was done by applying a version of the equation by Widmark [1] and Watson et al. [2] for males:

$$amount\ of\ alcohol\ in\ grams\ = \frac{TBW\ \times\ 1.055\ \times\ max.BrAC}{0.8}$$

where *TBW* is calculated as

$$TBW = 2.447 - (0.09516\ \times\ age) + (0.1074\ \times\ height) + (0.3362\ \times\ weight).$$

Group differences in experimental procedure and alcohol administration are detailed below.

Intoxication group

Intoxication appointments started on Friday or Saturday between 3 and 7 p.m.. Participants were asked to stop eating at least three hours prior to the intoxication appointment in order to induce the acute intoxication on a relatively empty stomach, as this reduces the resorption deficit, thus making rapid intoxication safer for the participants. Assuming a resorption deficit of 20 % on an empty stomach, an average BrAC of 1.2 ‰ can be achieved without exceeding the possible upper limit of 1.6 ‰ (which would have been reached at a resorption deficit of 0 %). To reach the goal of an average BrAC level of 1.2 ‰, we administered 1.98 grams of alcohol per liter of estimated TBW. This was converted into an individual amount of vodka (40 % alcohol by volume (ABV)) for each participant, which was diluted with the same amount of orange juice at room temperature. Mixtures of vodka and orange juice are considered the gold standard for orally induced acute intoxication, as this drink only contains very small amounts of congeners, thus reducing potential confounding factors, as well as the likelihood of next-day hangover [3]. The data sheet used to calculate individual alcohol amounts is provided as Supplementary Material. Participants were given 30 minutes to finish their individual beverage, and had another 30 minutes of waiting time before the experiment started. During the consumption and waiting time, participants could choose to either watch episodes of the television series "Shaun the Sheep" or the "Big Bang Theory" in order to standardize the entertainment during that time period and reduce the impact of variability of external factors during the waiting period on mood. Participants started the experimental task 30 minutes after the end of the alcohol consumption. BrAC was measured immediately before and after task completion. Afterwards, participants completed further functionally unrelated tasks, which are not part of this study, but have been published elsewhere [4,5]. Additional BrAC measurements were taken after each of these tasks as well, which was approximately 90 and 120 minutes after the end of alcohol consumption. Unlimited access to tap water was provided during and after the experimental task at both the sober and intoxicated appointment.

Hangover group

To induce hangover symptoms, participants joined an intoxication appointment at our facilities on the night before the hungover appointment. This intoxication appointment was scheduled on a Friday or Saturday evening (starting at 8 p.m. and finishing at around 1:30 to 2 a.m.). Participants were asked to partake on a full stomach to better cope with the administered alcohol amount and congener-rich alcohol type, as well as to reduce the likelihood of experiencing side effects like nausea, stomach pain and vomiting.

As the resorption deficit is higher on a full stomach (approx. 30-40 %), we administered 2.64 grams of alcohol per liter of estimated TBW. Given this assumed resorption deficit, participants were expected to achieve an average BrAC of 1.2-1.3 ‰ with only a very small likelihood of exceeding a BrAC of 1.6 ‰ (which would occur at a resorption deficit of 20 %, as typically observed on an empty stomach), and no possibility to exceed a max. BrAC of 2.0 ‰ (which would theoretically occur at a resorption deficit of 0 %). As an additional safety precaution, the consumption duration was extended to a minimum of 2 hours, which was controlled by the experimenters. Opposed to the vodka and orange juice mixture administered in the acute intoxication group, the hangover group was only served alcoholic beverages with a high congener content (e.g., brandy or red wine) as such beverages are more likely to provoke and lead to more intense hangover symptoms [3,6,7]. The individually calculated amount of alcohol was thus converted into amounts of brandy (36 % ABV) and/or red wine (9.5 % ABV). Both beverages were served by the experimenters in standardized portions of 200 mL red wine (15 gr of alcohol) or 50 mL brandy (14 g of alcohol) to ensure a constant quantity and speed of alcohol consumption across drinks. Experimenters furthermore recorded type and time of each issued drink for each individual participant. The data sheet used to calculate individual alcohol amounts and record consumption processes is provided as Supplementary Material. With each standardized drink, participants could choose between brandy and red wine, and whether to drink it pure, chilled on ice or mixed with decaffeinated coke, orange lemonade, or ginger ale. Snacks (chips and wine gums) and tap water were available at all times and their intake was not recorded. Given that smoking during alcohol consumption may increase the likelihood of occurrence and severity of hangover symptoms on the next morning [8], participants were allowed to smoke while drinking. This offer was taken by $n = 8$ participants, of which $n = 7$ stated to be regular smokers. BrAC was measured every 30 minutes after each participant's last sip of alcohol, that is 30, 60, 90 and 120 minutes after the end of consumption.
Hungover appointments started on Saturday or Sunday morning between 9 and 11 a.m. to slightly limit the amount of sleeping hours on the night before, as sleep duration seems to be negatively correlated with hangover severity [9]. To prevent a possible impact of residual ethanol in the blood, the hungover testing did not start until participants had reached BrAC readings of 0.00 ‰. After task completion, participants conducted further functionally unrelated tasks, which are not part of this study, but have been published elsewhere [10,11]. Unlimited access to tap water was provided during and after the experimental task at both the sober and hungover appointment.

References

1. Widmark, E. M. P. *Die Theoretischen Grundlagen Und Die Praktische Verwendbarkeit Der Gerichtlich-Medizinischen Alkoholbestimmung*. (Urban und Schwarzenberg, 1932).
2. Watson, P. E., Watson, I. D. & Batt, R. D. Total body water volumes for adult males and females estimated from simple anthropometric measurements. *The American Journal of Clinical Nutrition* **33**, 27–39 (1980).
3. Rohsenow, D. J. & Howland, J. The role of beverage congeners in hangover and other residual effects of alcohol intoxication: a review. *Curr Drug Abuse Rev* **3**, 76–79 (2010).

4. Bensmann, W., Zink, N., Werner, A., Beste, C. & Stock, A.-K. Acute Alcohol Effects on Response Inhibition Depend on Response Automatization, but not on GABA or Glutamate Levels in the ACC and Striatum. *JCM* **9**, 481 (2020).
5. Stock, A.-K., Bensmann, W., Zink, N., Münchau, A. & Beste, C. Automatic aspects of response selection remain unchanged during high-dose alcohol intoxication. *Addict Biol* e12852 (2019) doi:10.1111/adb.12852.
6. Rohsenow, D. J. *et al.* Intoxication With Bourbon Versus Vodka: Effects on Hangover, Sleep, and Next-Day Neurocognitive Performance in Young Adults. *Alcoholism: Clinical and Experimental Research* **34**, 509–518 (2010).
7. Verster, J. C. The alcohol hangover-a puzzling phenomenon. *Alcohol and Alcoholism* **43**, 124–126 (2008).
8. Jackson, K. M., Rohsenow, D. J., Piasecki, T. M., Howland, J. & Richardson, A. E. Role of Tobacco Smoking in Hangover Symptoms Among University Students. *J. Stud. Alcohol Drugs* **74**, 41–49 (2013).
9. van Schrojenstein Lantman, M., van de Loo, A., Mackus, M. & Verster, J. Development of a Definition for the Alcohol Hangover: Consumer Descriptions and Expert Consensus. *CDAR* **9**, 148–154 (2017).
10. Opitz, A., Hubert, J., Beste, C. & Stock, A.-K. Alcohol Hangover Slightly Impairs Response Selection but not Response Inhibition. *J Clin Med* **8**, (2019).
11. Opitz, A., Beste, C. & Stock, A.-K. Alcohol Hangover Differentially Modulates the Processing of Relevant and Irrelevant Information. *Journal of Clinical Medicine* **9**, 778 (2020).
